# Effect of varying skin surface electrode position on electroretinogram responses recorded using a handheld stimulating and recording system

**DOI:** 10.1007/s10633-018-9652-z

**Published:** 2018-07-25

**Authors:** Angharad E. Hobby, Diana Kozareva, Ekaterina Yonova-Doing, Ibtesham T. Hossain, Mohamed Katta, Byki Huntjens, Christopher J. Hammond, Alison M. Binns, Omar A. Mahroo

**Affiliations:** 1 Department of Ophthalmology, King’s College London, St Thomas’ Hospital Campus, London, SE1 7EH UK; 2 Centre of Applied Vision Research, City, University of London, Northampton Square, London, EC1V 0HB UK; 3 Department of Twin Research and Genetic Epidemiology, King’s College London, St Thomas’ Hospital Campus, London, SE1 7EH UK; 4 UCL Institute of Ophthalmology, University College London, London, EC1V 9EL UK; 5 Medical Retina Service, Moorfields Eye Hospital, 162 City Road, London, EC1V 2PD UK; 6 Department of Physiology, Development and Neuroscience, University of Cambridge, Cambridge, CB2 3EG UK

**Keywords:** Electroretinogram, Retina, Electrode, Retinal function

## Abstract

**Purpose:**

A handheld device (the RETeval system, LKC Technologies) aims to increase the ease of electroretinogram (ERG) recording by using specially designed skin electrodes, rather than corneal electrodes. We explored effects of electrode position on response parameters recorded using this device.

**Methods:**

Healthy adult twins were recruited from the TwinsUK cohort and underwent recording of light-adapted flicker ERGs (corresponding to international standard stimuli). In Group 1, skin electrodes were placed in a “comfortable” position, which was up to 20 mm below the lid margin. For subsequent participants (Group 2), the electrode was positioned 2 mm from the lid margin as recommended by the manufacturer. Amplitudes and peak times (averaged from both eyes) were compared between groups after age-matching and inclusion of only one twin per pair. Light-adapted flicker and flash ERGs were recorded for an additional 10 healthy subjects in two consecutive recording sessions: in the test eye, electrode position was varied from 2 to 10–20 mm below the lid margin between sessions; in the fellow (control) eye, the electrode was 2 mm below the lid margin throughout. Amplitudes and peak times (test eye normalised to control eye) were compared for the two sessions.

**Results:**

Including one twin per pair, and age-matching yielded 28 individuals per group. Flicker ERG amplitudes were significantly lower for Group 1 than Group 2 participants (*p *= 0.0024). However, mean peak times did not differ between groups (*p *= 0.54). For the subjects in whom electrode position was changed between recording sessions, flash and flicker amplitudes were significantly lower when positioned further from the lid margin (*p *< 0.005), but peak times were similar (*p *> 0.5).

**Conclusions:**

Moving the skin electrodes further from the lid margin significantly reduces response amplitudes, highlighting the importance of consistent electrode positioning. However, this does not significantly affect peak times. Thus, it may be feasible to adopt a more comfortable position in participants who cannot tolerate the recommended position if analysis is restricted to peak time parameters.

## Introduction

The RETeval™ (LKC Technologies, Inc., Gaithersburg, MD, USA) is a handheld device for recording the full-field electroretinogram (ERG), which aims to improve the ease of ERG recording in a general clinical or office environment [1–3]. Equipped with a small Ganzfeld dome, an infrared LED and camera system (for monitoring the subject’s eye) and skin electrodes, it is designed to record monocular ERGs. An inbuilt pupilometer measures pupil diameter and adjusts stimulus strength accordingly to achieve consistent retinal illuminance, allowing ERGs to be recorded with natural pupils.

**Fig. 1:**
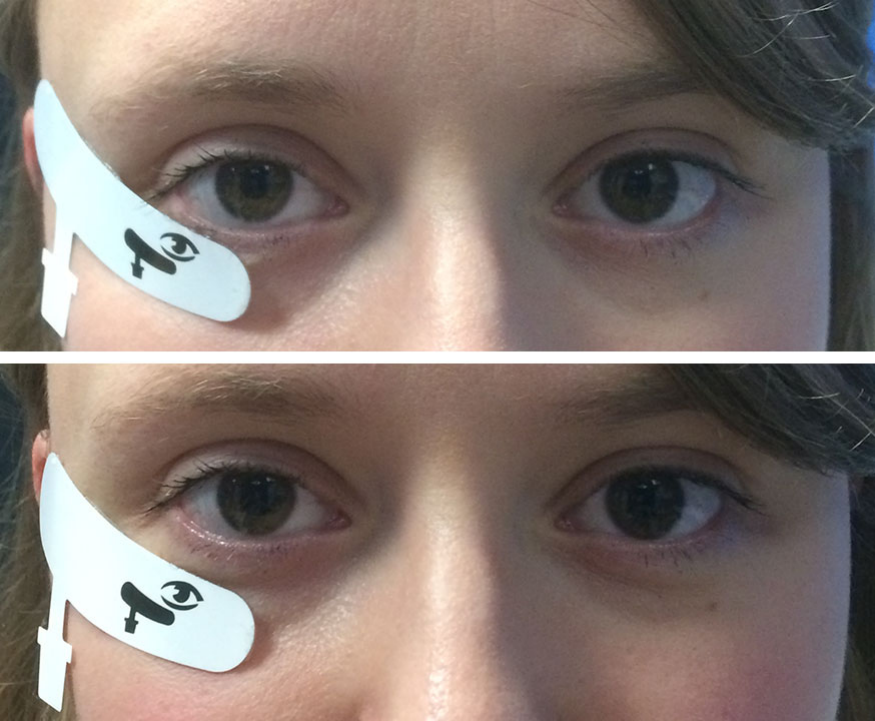
Two different electrode positions in a study participant. The skin sensor electrodes are placed 2 mm below the lid margin (manufacturer’s recommended position) in the upper photograph, and in an inferior position 10–20 mm below lid margin (lower photograph). Permission granted for use of photograph

To minimise invasiveness, the device is designed for use with skin surface “sensor strips”. These disposable, single use skin surface electrodes are specific to the right and left eye, respectively. The manufacturer recommends that the nasal edge of the sensor strip should be aligned with the midline of the pupil and the superior edge of the strip should be placed 2 mm below the patient’s lower lid margin (Fig. 1). This type of electrode generally requires little application time and has anecdotally proven to be more comfortable for patients undergoing ERGs than electrodes that make contact with the ocular surface. However, some subjects report mild discomfort with the recommended position due to proximity to eyelashes and interference with eye closure, and an alternative electrode position, further from the lid margin, over the orbital rim, can be more comfortable.

The use of periorbital skin surface electrodes to record the electroretinogram has a long history, first described in 1942 [4], and reported in several publications since (references [5–11] are a selection). Response amplitudes are substantially smaller than those recorded with corneal electrodes [8–11], and effects of different electrode positions, as well as directions of gaze, have been examined [5, 6, 9]: substantial changes in amplitude are observed in different recording positions.

Given the special design of the RETeval skin electrodes, incorporating active, reference and ground electrodes in a single strip, and their increasing use worldwide, we sought to examine, in this specific case, the effect of the different electrode positions on amplitudes and peak times of standard light-adapted responses (designed to correspond to ISCEV (International Society for the Clinical Electrophysiology of Vision) standard stimuli [12]) recorded using the RETeval stimulator.

## Methods

As part of a larger ongoing study, exploring genetic and environmental factors affecting ERG recordings in health and disease, adult twins were recruited from the TwinsUK cohort based at St Thomas’ Hospital London [13] and underwent recording of light-adapted flicker ERGs using the handheld stimulator. Participants gave informed consent; the study had ethics committee approval and adhered to the tenets of the Declaration of Helsinki.

Stimuli were delivered through natural pupils, and strengths were automatically adjusted by the device according to pupil diameter to correspond to international standard stimuli. The ISCEV standard specifies the delivery of 3.0 photopic cd m^−2^ s white stimuli in the presence of a 30 cd m^−2^ white background [12]. The device delivers 85 Td s stimuli [chromaticity (0.33, 0.33)] on a background of 850 Td (which would correspond to the ISCEV standard strength stimulus delivered through a 6-mm-diameter pupil). The white flicker and background stimuli were produced by simultaneous activation of red, green and blue LEDs. Flicker stimuli were delivered at 28.3 Hz, and the response was an average of 141–424 presentations. In most cases, each stimulus run was repeated once, and the parameters averaged. Participants were already light-adapted in standard indoor incandescent illumination (approximately 40–100 photopic cd m^−2^).

In the first 48 consecutively recruited participants (Group 1), skin electrodes were placed in a “comfortable” position, ranging from near the lid margin to approximately 2 cm below the lid margin. In the next 200 consecutive participants (Group 2), electrodes were positioned in the manufacturer recommended location (Fig. 1).

As ERG parameters from both twins of a given pair are highly correlated [14], only one twin from each pair was included for subsequent analysis. As ERG parameters vary with age [14, 15], the two groups were age-matched by ensuring the same numbers of participants in each age decade. This resulted in 28 participants per group. ERG parameters (flicker amplitudes and peak times), averaged from both eyes, were compared between the two age-matched groups (Mann–Whitney test).

In a third group of healthy participants (*n *= 10), photopic flash (85 Td s, 2 Hz) and flicker ERGs (as above) were recorded. Thirty flashes were delivered per run, and the series was repeated, so final parameters were typically the average from 60 consecutive flash presentations. The duration of each flash was less than 5 ms. The following protocol was undertaken in two consecutive recording sessions: in one session, electrodes were placed in the recommended position for both eyes; in the other session, the electrode was placed 10–20 mm below the lid margin in the test eye, but remained in the recommended position (2 mm below the lid margin) in the fellow eye. ERG parameters from the test eye were normalised to those of the control eye (test eye divided by control eye) to control for any adaptational changes between the two recording sessions, and the resulting normalised parameters compared between sessions (Wilcoxon signed rank test).

## Results

### Intergroup comparison

In the first group, 48 consecutive twin participants underwent recordings. Including only one twin per pair yielded 28 participants, mean (SD) age 60.3 (10.0) years (median 59.0; minimum 43.2; maximum 76.6; interquartile range 17.1 years); all were female (reflecting the demographics of the TwinsUK cohort which is overwhelmingly female). In the second group of 200 consecutive participants, including only one twin per pair gave 122 participants, mean (SD) age 46.7 (16.8) years; 14.7% were male. The mean ages were significantly different (*p *< 0.0001). The cohorts were then age- and sex-matched by first removing the males from the second group, and then including the same numbers of participants per decade as in the first group (by excluding any excess participants in each decade in the second group in chronological order of recruitment). The mean age of the remaining 28 participants in the second group was now 61.0 (9.4) years (median 63.3; minimum 43.4; maximum 77.5; interquartile range 16.7 years), which was not significantly different from Group 1 (*p *= 0.805).

**Fig. 2:**
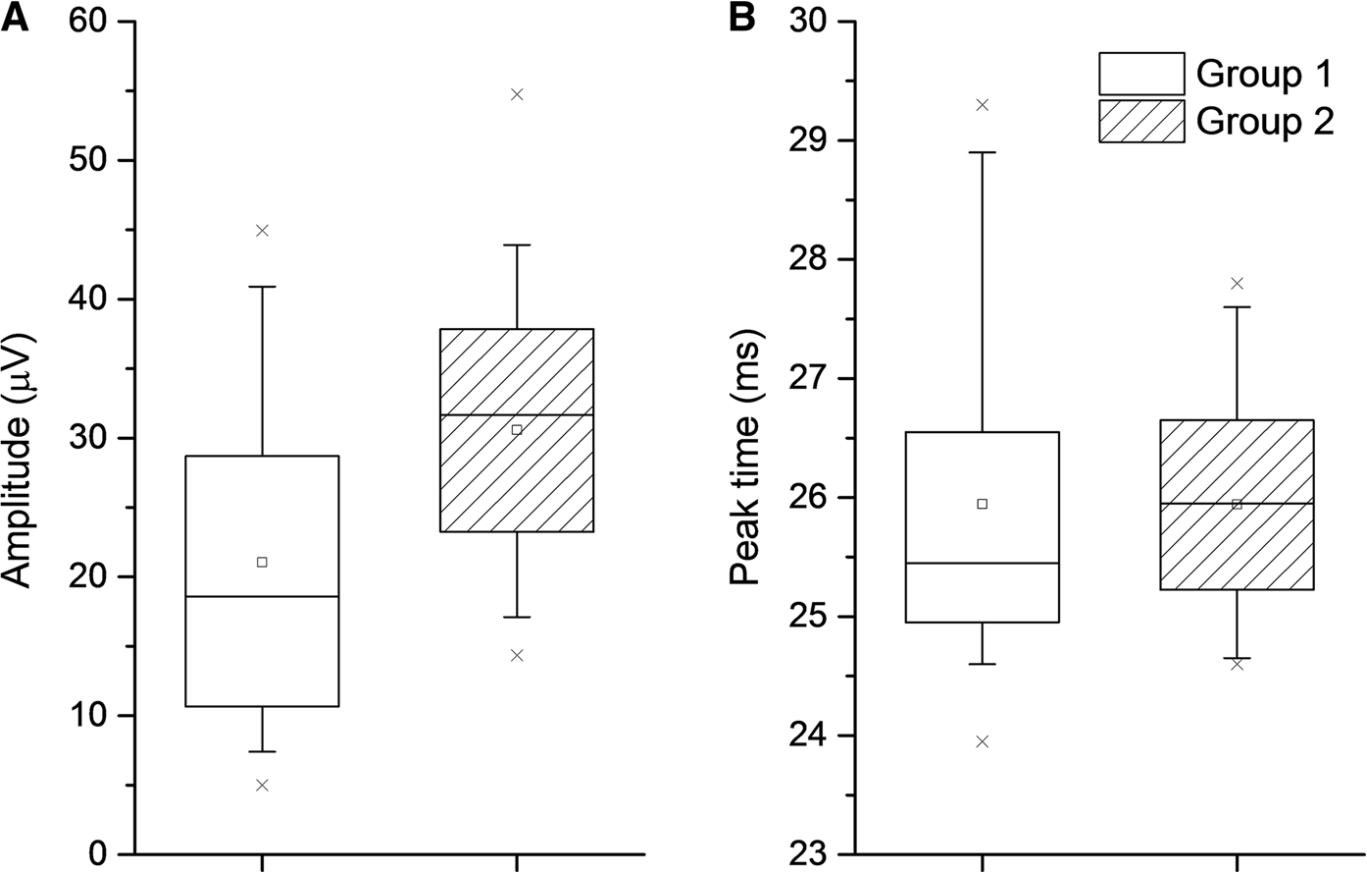
Boxplots comparing the distribution of ERG parameters between participants in Groups 1 and 2. In Group 1, the electrode was in a comfortable position (up to 20 mm from the lid margin); in Group 2, the electrode was placed in the recommended position of 2 mm below the lid margin. Median, upper and lower quartiles (limits of box) are shown. Squares plot means; whiskers span the 5th–95th centiles; and crosses plot minimum and maximum data points. Amplitudes are plotted in **a** and peak times in **b**

Table 1 compares flicker amplitudes and implicit times for the two groups. Amplitudes were significantly lower in Group 1 compared with Group 2 (*p *= 0.0024) whilst peak times did not differ significantly (*p *= 0.54). Figure 2 compares the distribution of values between the two groups as boxplots. The median amplitude was 41% lower for Group 1 compared with Group 2. If right and left eyes were compared separately, the same pattern was seen: amplitudes were significantly lower in Group 1 (*p *= 4.8 × 10^−4^ and 0.012 for right and left eyes, respectively), whilst peak times were not significantly different (*p* = 0.90 and 0.18 for right and left eyes, respectively).

**Table 1:** Parameter values compared between the two age-matched groups

	Group 1 (“comfortable” electrode position)		Group 2 (recommended electrode position)		*p* value for comparison (Mann–Whitney)
	Mean (SD)	Median (LQ, UQ)	Mean (SD)	Median (LQ, UQ)	
Flicker ERG amplitude (microvolts)	21.0 (12.0)	18.6 (10.7, 28.7)	30.6 (9.7)	31.7 (23.3, 37.9)	0.0024*
Flicker ERG peak time (ms)	25.9 (1.4)	25.5 (25.0, 26.6)	25.9 (0.9)	26.0 (25.2, 26.7)	0.54

*p < 0.05 regarded as significant

If, instead of age-matching the groups, parameters were compared between the two electrode positions adjusting for age as a covariant (28 participants from the first group, and 117 from the second group in whom measurements from both eyes were available), amplitudes were again found to be lower in the first group, but peak times were similar (*p *= 0.0001 and 0.36 for averaged amplitude and peak time comparisons, respectively).

### Intra-subject comparison

**Fig. 3:**
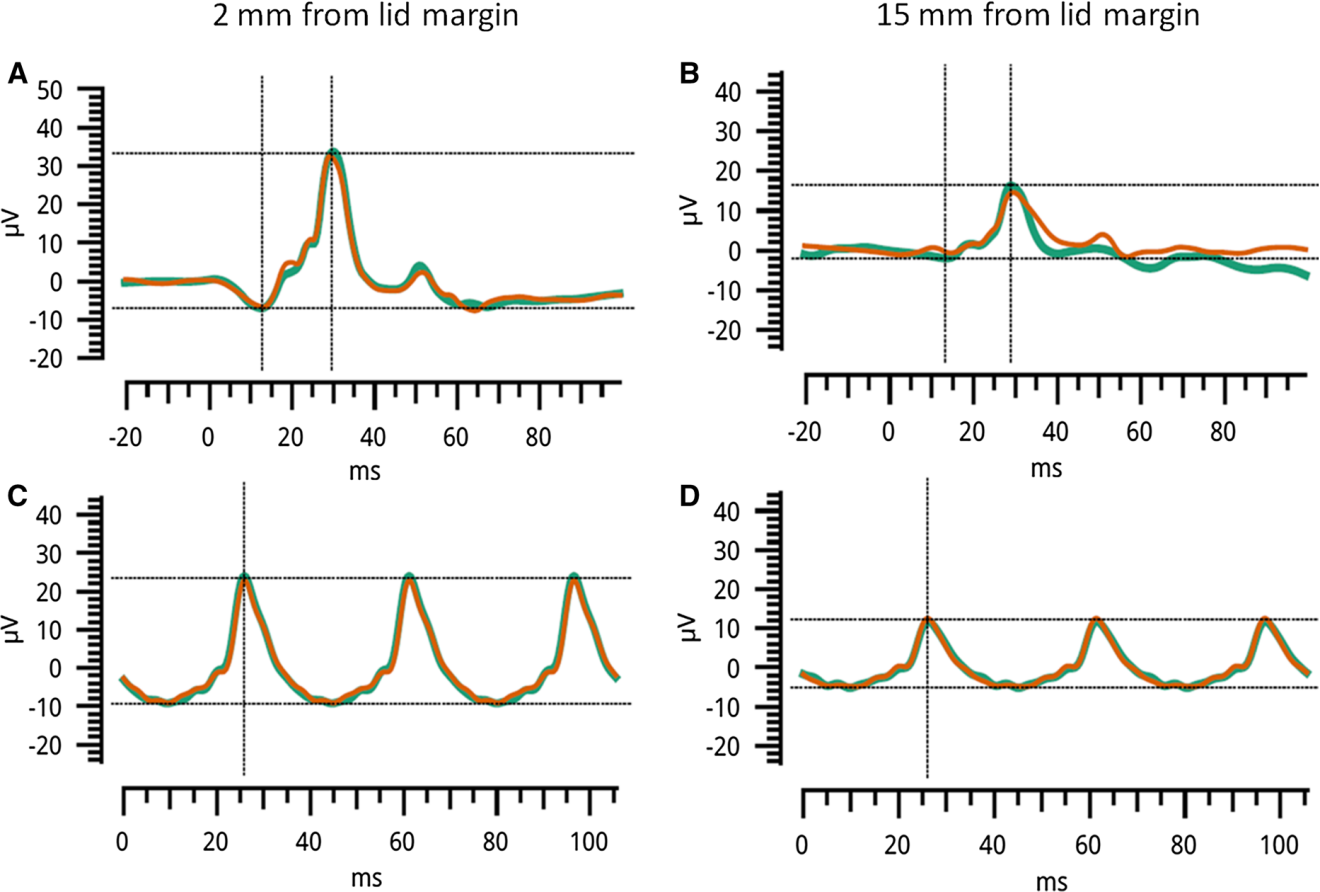
Sample traces from one participant. **a**, **b** Averaged light-adapted ERGs to a flash obtained from right and left eyes, respectively. In the right eye, the skin electrode was placed 2 mm from the lid margin. In the left eye, the electrode was 15 mm below the lid margin. **c**, **d** Light-adapted responses to the flicker stimulus obtained with electrodes placed as in **a**, **b,** respectively. Green and orange traces represent averaged recordings obtained from consecutive runs of stimulus presentations

**Fig. 4:**
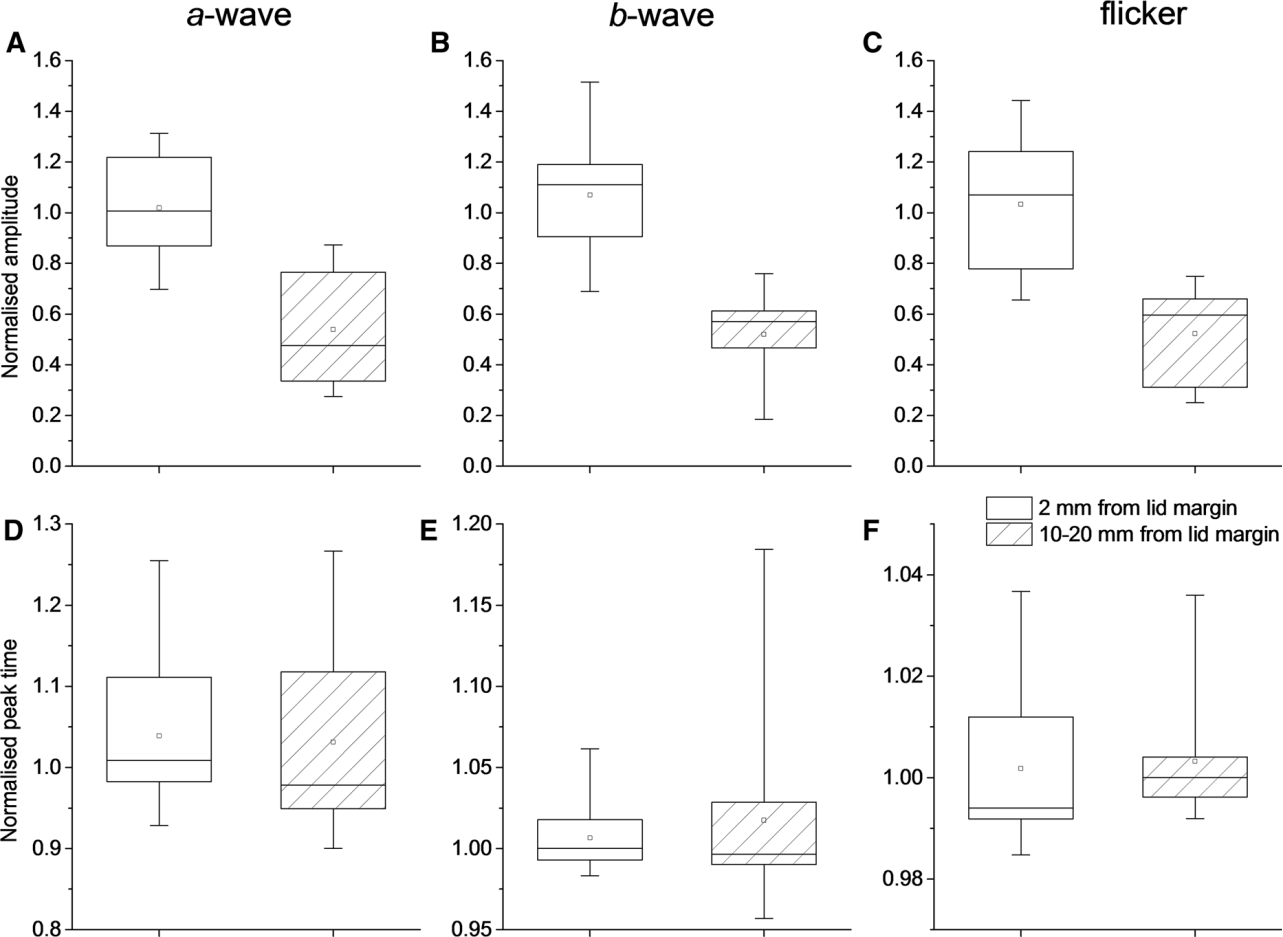
ERG parameters for different electrode positions in participants in the third group (*n *= 10; data normalised to control eye). **a**, **b**, **c** Boxplots showing a-wave, b-wave and flicker ERG amplitudes: differences were significant between the two positions (*p *= 0.004, 0.002 and 0.002, respectively, Wilcoxon signed rank test). **d**, **e**, **f**, Boxplots showing a-wave, b-wave and flicker ERG peak times: differences were not significant (*p *= 0.85, 0.57 and 0.56, respectively). Boxes show median and upper and lower quartiles; squares plot mean values; whiskers extend to maximum and minimum values. Note that the *y*-axis scales differ in the lower panels and have been expanded considerably in *F*, where the actual range between maximum and minimum values is less than 0.1

In the second part of the study, 10 healthy volunteers (4 male; 6 female) underwent consecutive recordings [age range 22.9–45.3 years; mean (SD) 33.1 (7.1) years; median 34.2 years]. Sample recordings from one participant are shown in Fig. 3: the waveforms were similar in both recording positions, but the response was substantially larger in the lid margin position. Green and orange lines show averages of consecutive runs of stimulus presentations, showing reasonably good intra-session reproducibility. Figure 4 shows distributions for amplitudes and peak times for the group (test eye normalised to fellow eye as described in “Methods”). Again, amplitudes were significantly lower when the electrode was 10–20 mm, compared with 2 mm, from the lid margin, but peak times were similar; *p* values are given in the figure legend. Median amplitudes were 44–53% lower in the second position compared with the first.

## Discussion

This study investigated the effect on the ERG of moving specially designed skin electrodes further inferiorly from the lid margin, both by comparing light-adapted flicker ERGs in two large groups of participants (with the two groups differing by electrode position), and by recording light-adapted flash and flicker ERGs in a smaller group in whom recordings were performed using two different positions in consecutive sessions. The findings from both investigations were consistent: placing the electrode in a more inferior position resulted in a significant reduction in response amplitudes (by approximately 40–50%) although peak times appeared to be unaffected. This finding emphasises the importance of consistent skin electrode positioning, particularly when amplitudes are being analysed.

The proportionate change in median amplitude for the intra-subject comparison (44–53% reduction when further from the lid margin) is broadly similar to that seen in the intergroup comparison (41%), though slightly higher. The “comfortable” position varied substantially in both comparisons (whilst the position 2 mm from the lid margin was consistent), and it is possible that this was more frequently closer to the lid margin in the intergroup comparison than in the intra-subject comparison.

Previous studies of skin electrode position have shown a reduction in amplitude the further the recording electrode is from the cornea [5, 6, 9]. Also, we have found that ERGs recorded with conductive fibre electrodes placed in the fornix are substantially lower in amplitude than those recorded with the electrode at the lid margin, whilst peak times are similar (Tariq et al. [16], ARVO meeting abstract 5121). This result was consistent with the findings of a similar study of conductive fibre electrode position recently published, showing a 20–25% reduction in amplitude [17]. Overall, the present study fits well with the findings of previous studies of both skin and corneal conductive fibre electrodes: the further the recording electrode is from the corneal apex, the lower the recorded signal amplitude.

The findings of the present and previous studies suggest that skin recording electrode positioning is less critical with respect to analysis of peak time parameters. Nevertheless, the electrode must be positioned sufficiently close to the eye to elicit an ERG in which the peaks and troughs are discernible. In this study, all healthy subjects produced a measurable flicker ERG, even when the electrode was positioned up to 20 mm from the lid margin. However, in patients with subnormal responses in retinal disease, this can be more challenging. In some patients with reduced cone system responses measured using conventional ERG recording systems, it has not been possible to accurately determine peak times using the RETeval system as amplitudes are too low to distinguish response components [3].

One limitation of the present study was that electrode placement in Group 1 was not consistent and was determined more by participant “comfort”, whilst positioning in Group 2 was consistent. Also, the participants were not randomised to different electrode positions, but the position was determined by whether they were in the first or second phase of subject recruitment. However, recordings from the third group of participants (in whom both positions were used in the same subject) were consistent with the findings from the first two groups.

Also, potential time-dependent adaptational effects (the right eye was recorded from first in all subjects) were controlled for, in the first two groups, by averaging both eyes, and, in the third group, by normalising the test eye to the control eye. Even when right and left eyes were compared separately in the first two groups, the results were found to be similar (amplitudes differed significantly between the two positions, but peak times did not).

Although multiple parameters were compared in the intra-subject comparison, a correction for multiple testing was not deemed necessary [18]. The comparisons were predetermined and essentially explored differences in only two types of parameter, namely amplitudes and peak times. The finding that amplitudes were significantly different, and peak times were not, was observed consistently across all parameter comparisons, strongly supporting the validity of the conclusions of this study.
